# Therapeutic use of psilocybin: Practical considerations for dosing and administration

**DOI:** 10.3389/fpsyt.2022.1040217

**Published:** 2022-12-01

**Authors:** Caroline A. MacCallum, Lindsay A. Lo, Carly A. Pistawka, Jagpaul Kaur Deol

**Affiliations:** ^1^Department of Medicine, Faculty of Medicine, University of British Columbia, Vancouver, BC, Canada; ^2^Department of Public Health Sciences, Dalla Lana School of Public Health, University of Toronto, Toronto, ON, Canada; ^3^Faculty of Science, University of British Columbia, Vancouver, BC, Canada; ^4^Faculty of Pharmaceutical Sciences, University of British Columbia, Vancouver, BC, Canada

**Keywords:** psilocybin, psilocybin-assisted psychotherapy, psychedelics, psilocin, magic mushrooms, patient safety

## Abstract

The interest in psilocybin as a therapeutic approach has grown exponentially in recent years. Despite increasing access, there remains a lack of practical guidance on the topic for health care professionals. This is particularly concerning given the medical complexity and vulnerable nature of patients for whom psilocybin-assisted psychotherapy may be considered. This article aims to provide health care professionals with an overview of practical considerations for psilocybin therapy, rooted in a patient safety focus. Within this piece we will review basic psilocybin pharmacology and pharmacokinetics, indications, practical therapeutic strategies (e.g., dosing, administration, monitoring) and safety considerations (e.g., contraindications, adverse events, and drug interactions). With this information, our goal is to increase the knowledge and comfort of health care professionals to discuss and counsel their patients on psilocybin therapy, ultimately improving patient care and safety.

## Introduction

The interest in psilocybin as a therapeutic approach has grown exponentially in recent years. Primarily originating from fungal species within the genus *Psilocybe*, psilocybin is an indole alkaloid that is the main psychedelic ingredient in psychedelic mushrooms (1). *Psilocybe* mushroom species are pan-tropical, growing around the globe, including in the regions of the southeastern United States, Central and South America, South East Asia, and parts of Africa (2, 3). Although interest in psychedelics, and more specifically psilocybin, has emerged relatively recently within western culture, the traditional and ancestral use of psychedelic mushrooms originated generations ago in Mesoamerica (1, 4, 5). Civilizations such as the Aztec, Maya, Olmec, and Zapotec have documented use of psilocybin to evoke altered states of consciousness for healing rituals and religious ceremonies (4). In recent years, psilocybin has gained traction as a potential therapeutic agent within western health care. Several high profile trials have shown promising results for end of life distress and treatment-resistant depression (6–10).

Access to psilocybin worldwide has been largely restricted since the 1960’s (11). Richard Nixon’s “war on drugs” combined with tighter regulation of pharmaceutical research is largely responsible for the halting of psychedelic research and subsequent restricted access for therapeutic purposes in North America (11, 12). Despite support for the safety and efficacy of psilocybin and other psychedelics (13), research and exploration of psilocybin as a therapeutic has not re-emerged until recently.

The US Food and Drug Administration granted breakthrough therapy status to psilocybin in 2018 for treatment-resistant depression, and in 2019 for major depressive disorder. At a state-level, Oregon has more recently passed Ballot Measure 109 allowing for the manufacture, delivery, and administration of psilocybin within a to-be-developed state-run program – an initiative is being paralleled by efforts at other local jurisdictions (e.g., Denver).

In Canada, psilocybin possession is illegal except through Health Canada-approved pathways: research (including clinical trials), Section 56 exemption, and the Special Access Program (SAP). Both Section 56 exemptions and SAP allow for limited medical use of psilocybin outside of research settings if it is believed to be necessary for medical purposes (14, 15). Prior to 2022, Section 56 exemptions were the sole option, but this route was flawed due to: no access to legal/safe supply of psilocybin (patients had to source non-good manufacturing practice (GMP) psilocybin themselves through illicit sources), limited approvals being granted, lack of transparency on denials from Health Canada, long wait times of up to 300 days for approvals, and many exemption requests left unanswered (15). A primary pathway was created following a 2022 SAP amendment, where SAP submissions receive responses within 24–48 h and, if approved, patients can procure regulated psilocybin from Health Canada licensed dealers. Section 56 exemptions have now become a secondary pathway to be used after a denial is received for a SAP request (16).

As access and public awareness to psilocybin increases, it is prudent for all health care professionals (HCP) to have a baseline pharmacotherapy knowledge of this treatment option. Understanding and applying patient specific safety considerations is essential in assessing psilocybin eligibility and appropriately managing patient care, even if a HCP is involved indirectly. Furthermore, due to the movement of global jurisdictions toward decriminalization of various psychedelic substances, discussions on personal use (with or without therapeutic intent) may also become a part of primary care, similar to what has happened with cannabis.

There remains a lack of practical clinical guidance for HCPs on Psilocybin-assisted psychotherapy (PAP). This is particularly concerning given the complex and medically vulnerable nature of patients who may qualify for this treatment modality. As such, this article aims to provide HCPs with an overview of the practical considerations for PAP that can be utilized when considering, counseling, prescribing, or monitoring psilocybin use in a patient. Although this paper focuses on the medical use and access channels to psilocybin, we acknowledge and support that non-medical model access needs to be established as there exists a spectrum of use that extends beyond prescriptive medical access.

## Pharmacology in brief

Psilocybin (4-phosphoryloxy-N,N-dimethyltryptamine) and its pharmacologically active metabolite psilocin (4-hydroxy-N,N-dimethyltryptamine) are the major psychoactive alkaloids in several species of “magic” mushrooms (17, 18). They are tryptamine/indolamine hallucinogens, structurally related to serotonin (17). Psilocybin and psilocin both display non-specific partial agonist activity on the serotonergic neurotransmitter system, with varying binding affinities at several serotonergic receptor sites (19, 20).

Psilocin, being highly lipophilic, is able to cross the blood-brain barrier and bind to several serotonergic receptors with a particularly high binding affinity to 5-hydroxytryptamine 2A (5-HT_2*A*_) receptor as compared to psilocybin which is hydrophilic and cannot readily cross the blood-brain barrier (17, 21–23). As with all classical tryptamine psychedelics, the subjective effects of psilocin are mediated by biased (functionally selective) agonism of 5-HT_2*A*_ receptors (24). Psilocin binding to the 5-HT_2*A*_ receptor creates functional selectivity which favors the psychedelic signaling pathway over the default serotonin pathway (22, 24–26). The downstream signaling bias, as a result of functional selectivity, leads to increased glutamate release which also likely contributes to the psychedelic effects of psilocin (24, 27). Additionally, it is proposed that psilocin’s neurobiological signaling pathways induce changes in neuroplasticity through (but not limited to) increased expression of glutamate and brain-derived neurotrophic factor (BDNF) (28, 29). The potential connection between BDNF and depression (30, 31) is one suggested mechanism for psilocin’s therapeutic effect (28, 29).

5-HT_2*A*_ receptors are also highly expressed in the visual cortex, contributing to the visual hallucinations associated with psilocin (27, 32). Antagonism of these receptors using the 5-HT_2*A*_ receptor blocker ketanserin has shown attenuation of hallucinatory effects, supporting the underlying psychedelic mechanism of action through 5-HT_2*A*_ receptors (33–35). Additional binding sites, including 5-HT_1*A*_ and 5-HT_2*C*_, may potentially play a role in some of the psychedelic effects of psilocin, but further studies are needed to determine the extent of this contribution (20, 22, 24).

## Pharmacokinetics

Psilocybin is a prodrug and must be converted to its metabolite psilocin in order to cross the blood-brain barrier and elicit its neurological effects. Following oral ingestion, psilocybin is rapidly dephosphorylated via the acidic environment of the stomach into psilocin. Any remaining psilocybin is then converted in the intestines, blood, and kidneys through alkaline phosphatase to produce the active, lipophilic form, psilocin (17, 23). It is important to note that the majority of psilocybin efficacy and pharmacokinetic studies are based on patients fasting for an average of 2-4 h (except water). In order to ensure more predictable kinetics, good clinical practice should include instructions for consuming psilocybin on an empty stomach.

**FIGURE 1:**
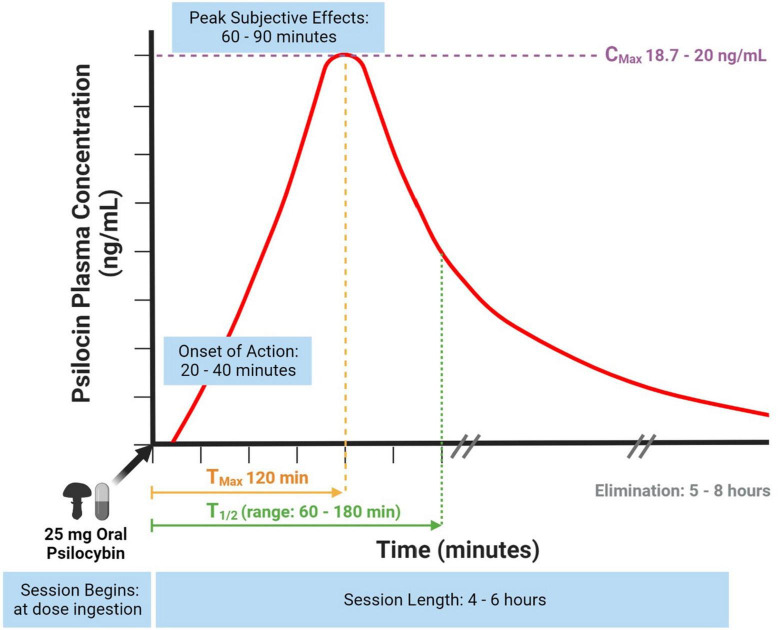
Psilocin concentration to session time curve using a standard psilocybin dose. Information gathered from (17, 18, 23, 28, 36, 37).

The bioavailability of psilocybin is approximately 50% following oral ingestion (17). Psilocybin is a water-soluble compound detectable in plasma within 20–40 min (Figure 1) (17). Psilocin is extensively distributed through the bloodstream to all tissues, including crossing the blood-brain barrier to elicit central nervous system effects (23). Psilocin is detectable in plasma after 30 min (17) and displays linear pharmacokinetics (36).

In earlier studies, the half-life (T_1/2_) of psilocin was found to be 50 min and T_1/2_ of psilocybin being 160 min (17) whereas newer studies using a fixed 25 mg psilocybin dose and specifically measuring unconjugated psilocin found its T_1/2_ to be 108 min (range: 66–132 min) (Figure 1) (28). The dose response curve correlates to a session experience with an onset of action at 20-40 min, peak subjective effects at 60–90 min and an active duration of 4–6 h (Figure 1) (18). A dose-escalating study of oral psilocybin found an average psilocin T_1/2_ of 3 h, but also found minor variability (standard deviation 1.1) between participants (Figure 1) (36). Variability was not predicted by body weight, and may instead be due to less or more hydrolysis of the psilocin glucuronide metabolite (36). After a 25 mg fixed oral dose of psilocybin, one study reported a maximal psilocin plasma concentration at 120 min (Tmax) of 20 ng/mL (Cmax) (28) and similarly, another study reported a Cmax of 18.7 ng/mL (37).

A recent study shows that ∼80% of all circulating psilocin is metabolized by hepatic phase II glucuronidation (conjugation) through UGT 1A10 and UGT 1A9 enzymes into psilocin-O-glucuronide (23). This inactive secondary metabolite is renally cleared, along with ∼2% of combined unconjugated psilocin and unmetabolized psilocybin (36). The remaining ∼20% of circulating psilocin is metabolized through several pathways (including MAO, ALDH, cytochrome oxidase, etc.) and excreted through the bile into the stool (23). Complete psilocin excretion occurs within 24 h, with the majority occurring in the first 8 h (17, 23).

## Therapeutic uses

The strongest evidence for psilocybin is from clinical trials in cancer-related depression and anxiety and treatment-resistant depression (Table 1) (7–10, 38–41). Alcohol use disorder and tobacco addiction have been found to have moderate-level evidence from several clinical trials (42–45).

**TABLE 1:** Evidence for psilocybin-assisted psychotherapy.

Least evidence^a^	Some evidence^b^	Most evidence^c^
Obsessive Compulsive Disorder (46) Cluster Headaches/Migraines (47, 48) Demoralization with AIDS Diagnosis (50)	Alcohol Use Disorder/Dependence (42–44) Tobacco Addiction (45)	Cancer-related depression (8–10) Cancer-related anxiety (8–10) Treatment-resistant depression (7, 38, 39)

Evidence definitions:

^a^Least Evidence: case reports, case series, lack of clinically significant results.

^b^Some Evidence: single group, open label trials, proof-of-concept trials.

^c^Most Evidence: multiple clinical randomized controlled trials (RCTs).

Preliminary research (e.g., proof-of-concept, open label trials, or case series) suggests there may also be utility for psilocybin in obsessive compulsive disorder (OCD) (46), cluster headaches (47, 48), migraines (49), and demoralization with an AIDS diagnosis (50). Currently, clinical trials are being conducted on a wide range of conditions including migraines, addiction (tobacco, alcohol, cocaine), anorexia, bipolar disorder, chronic pain, major depressive disorder, and OCD (51).

## Dosing and administration strategies

Many earlier psilocybin trials initially dosed using body weight, with doses generally ranging from 0.2–0.4 mg/kg of psilocybin per session. Protocols consisted of either a single dose or two doses separated by an average 3–4 week washout period. Current clinical trial protocols have switched to using a fixed psilocybin dose, most commonly 25 mg, which is consistent with the previous 0.3 mg/kg weight-based dosing (36). The fixed 25 mg dose approach has been validated in a recent secondary analysis of prior trial data, which found no significant differences for psychedelic effects compared to weight-based doses of 0.29 mg/kg and 0.43 mg/kg (52). As the renally cleared secondary metabolite (psilocin-O-glucuronide) is inactive, it suggests that patients with mild-moderate renal impairment do not require a dose reduction (36). Table 2 compares several dosing ranges for both psilocybin and dried *Psilocybe cubensis* mushroom (a common research species) based on the above studies that can inform dosing guidelines.

**TABLE 2:** Psilocybin dosing and considerations.

	Standard dose	High dose	Supra-therapeutic dose
Psilocybin (extracted isolate or synthetic)	25 mg	35 mg	50–60 mg
Dried *Psilocybe cubensis* mushroom (whole or powdered) equivalent	2.5 gram*	3.5 gram*	5–6 gram*

*Conversions assume a **1% psilocybin per 1 gram** of dried *Psilocybe cubensis* mushroom. Psilocybin content between species and strains can range from 0.5–2% per gram. Information gathered from (36, 52, 73, 74).

Outside of research or medical access settings (such as Health Canada SAP), a common source of psilocybin is from dried “magic” mushrooms. This requires a conversion to determine the estimated weight of dried mushrooms to consume in order to arrive at the intended psilocybin dose. Based on several studies, the average psilocybin content is ∼1% psilocybin per one gram of dried *Psilocybe cubensis* mushroom; therefore, a 25 mg psilocybin fixed dose is approximately 2.5 grams of dried *Psilocybe cubensis* mushroom. However, it is important to note, there is intra- and inter-species variability of psilocybin content (53, 54). In psilocybin-naive patients using dried mushrooms, it is good clinical practice to start at a lower dried mushroom weight in the event that the actual psilocybin content is higher in any given batch. The variability in psilocybin content can range on average from 0.5–2% dried mushroom weight based on species (53, 54).

Underlying causes for varying sensitivities to psilocybin effects are not fully understood. Lab research in cells points to differences in 5-HT_2*A*_ receptors through single nucleotide polymorphism variants, such as Ala230Thr, as one factor (55). Clinically, these varying sensitivities are observed with a lack of fasting and certain conditions such as reduced gastric acid, altered gastric motility, or liver dysfunction.

The administration of psilocybin should be accompanied with assisted psychotherapy to maximize potential benefits. Psilocybin-assisted psychotherapy focuses on three stages: pretreatment, treatment, and posttreatment (56). Pre-treatment heavily focuses on building therapeutic rapport and trust (56). One fundamental therapeutic concept is that of “set and setting” which refers to appropriate patient preparation so that they are informed on what to expect during their session both from a mindset and environmental perspective. Treatment sessions are generally conducted in a non-directive, supportive setting and utilize a variety of tools such as a music playlist, meditation, and breath work (56, 57). Posttreatment focuses on supporting the integration of experiences (56). Detailed practical guidance on assisted-psychotherapy is out of scope for the present manuscript, however, it has been noted in a variety of other publications [e.g., (56–60)].

## Contraindications

Most contraindications are due to increased risk for *psychological* distress, including the rare, but potential, serious adverse event of psychosis (6–8, 38, 39, 61). History of schizophrenia, psychosis, bipolar disorder, and borderline personality disorder are generally contraindicated for psilocybin (6–8, 38, 39, 61); however, future observational data in these conditions may provide a more detailed risk-benefit. Pregnancy and breastfeeding are also contraindicated given insufficient scientific evidence to assess risk.

The primary *physical* concerns are due to transient increases in heart rate and blood pressure following psilocybin ingestion (8, 62). Thus, serious or uncontrolled cardiovascular conditions are often considered to be relative contraindications (39, 61).

## Drug interactions

The concurrent use of psychotropic medications, especially antidepressants and antipsychotics, may introduce risks (safety concerns) or alter benefits (efficacy concerns). Many of these medications modulate the serotonin system including 5-HT_2*A*_ receptors (61). As psilocybin and psilocin mainly interact with the serotonergic system, particularly 5-HT_2*A*_, there is a risk for pharmacodynamic drug interactions (17).

The most common pharmacodynamic interaction may result in a heightened or blunted psychedelic experience. For example, tricyclic antidepressants (TCAs) may increase the intensity, while monoamine oxidase inhibitors (MAOIs) and selective serotonin reuptake inhibitors (SSRIs) may decrease the intensity (61, 63–67).

Drug interactions between serotonergic drugs may lead to a rare, but serious, condition called serotonin syndrome in which excessive serotonin signaling can lead to a potentially life-threatening adverse drug reaction (68, 69).

Clinical trial protocols often require the tapering and washout of these medications due to the concern of serotonin syndrome, however, there is a lack of published serotonin toxicity cases. A recent standard dose psilocybin study of patients on a concurrent SSRI were found to have no additional safety concerns such as QT prolongation (an abnormally extended interval between heart contracting and relaxing), serotonin syndrome and no reduction in efficacy such as impact to positive mood (28). This continues to be an area of further investigation.

In addition, potential pharmacokinetic drug interactions exist for phase II UGT substrates. As psilocin is primarily metabolized by UGT 1A10 and 1A9 (23), medications that can inhibit or induce these enzymes must be held or tapered prior to administration of psilocybin. Some examples of UGT 1A10/1A9 inhibitors include diclofenac (a Non-steroidal anti-inflammatory drug) and probenecid (a uric acid reducer).

A detailed medication screening should be completed, especially for antidepressants, antipsychotics, psychostimulants, lithium, and other dopaminergic or serotonergic agents (39, 42, 61). If there is concern for drug-drug interactions, an individualized risk-benefit assessment should be conducted with an appropriate drug taper schedule if warranted. Health care professionals should be aware of potential discontinuation syndrome and monitor for signs of distress if tapering psychotropic medications prior to psilocybin administration.

## Adverse events

Due to psilocybin’s large therapeutic index (1:1000) and a typically unattainable lethal dose, psilocybin has a favorable safety profile (6, 9, 10, 42, 61, 62, 70, 71). Relative to other psychedelics (such as MDMA, DMT, etc.), psilocybin has lower occurrence of seizures, hospital admissions and other serious adverse effects, and lacks addictive or reinforcing properties (62, 72). Several dose-escalating studies have tested the subjective psychedelic effects in supratherapeutic doses, e.g., 50–60 mg or 5–6 grams, and found positive results with little-to-no safety concerns (36, 37, 73).

The primary risk for psilocybin is psychological safety, not physiological safety as it is for most classic drugs (e.g., opioids, sedatives, stimulants) (61, 69). Properly conducted PAP aims to minimize the potential for psychological harms through appropriate patient preparation and close patient monitoring. Despite this, the acute psychotomimetic effects associated with psilocybin may still pose a risk for psychological distress, and in rare cases, psychosis (61, 69). Physical and psychological adverse events are reported in Table 3. Many of these are transient in nature and related to the therapeutic nature of emotional processing in session (blood pressure, heart rate, anxiety). Transient headaches may also arise the day after treatment. Data from clinical trials, in which proper screening is completed and consumption is monitored, report no serious adverse events (6, 9, 10, 38, 39, 42, 46, 71).

**TABLE 3:** Psilocybin-associated adverse events.

Physical	Psychological
**Commonly reported adverse events**	
Increase in Blood Pressure and Heart Rate Headache Nausea	Anxiety Confusion
**Less commonly reported adverse events**	
Fatigue Migraine Vomiting Physical Discomfort	Strong or Extreme fear Paranoia Psychotic-like symptoms Psychological discomfort

Information gathered from (6, 10, 38, 39, 42, 45, 46, 69, 71).

## Conclusion

As interest and access to psilocybin as a therapeutic option grows, HCPs require sufficient information to navigate potential psilocybin use in their patients. At a time where the use of psilocybin is becoming more common, it is imperative for HCPs to have a base-level understanding of psilocybin, its indications, and key safety considerations in order to guide and counsel their patients. These factors also play an important role in informing policymakers as they create regulations and guidance for psilocybin use. Development of health care education in order to equip HCPs with the necessary knowledge to ensure patient safety are worthwhile goals.

## Author contributions

CM contributed to conception of manuscript. All authors wrote sections of the manuscript, contributed to manuscript revision, read, and approved the submitted version.
